# Height, weight, and alcohol consumption in relation to the risk of colorectal cancer in Japan: a prospective study

**DOI:** 10.1038/sj.bjc.6600845

**Published:** 2003-04-01

**Authors:** N Shimizu, C Nagata, H Shimizu, M Kametani, N Takeyama, T Ohnuma, S Matsushita

**Affiliations:** 1 Department of Epidemiology and Preventive Medicine, Gifu University School of Medicine, 40 Tsukasa-machi, Gifu 500-8705, Japan; 2 Department of Internal Medicine, Takayama Red Cross Hospital, 3-11 Tenma-cho, Takayama 506-8550, Japan; 3 Department of Internal Medicine, Takayama Kumiai Hospital, 5-68 Ojin-machi, Takayama 506-8502, Japan

**Keywords:** colorectal cancer, height, body mass, Japanese

## Abstract

Colorectal cancer incidence in relation to body size, smoking, and alcohol consumption was studied in a cohort of 29 051 city residents of Japan. In 1992, each participant completed a self-administered questionnaire on sociodemographic characteristics, drinking, cigarette smoking, diet, exercise, and reproductive and medical histories. The response rate was 92%. From 1993 to 2000, 161 men and 134 women were diagnosed with colorectal cancer at two major hospitals in the city. Relative risks and 95% confidence intervals were calculated by using Cox proportional hazard models. A positive relation between height and colorectal cancer was seen in both sexes, controlling for age, body mass index (BMI), smoking and drinking habits, and years of education. The findings were statistically significant only for men (relative risk 2.13 for the tallest compared with the shortest height tertile; 95% confidence interval=1.26–3.58). Body mass index was also associated positively with colon cancer risk for men, whereas the pattern for women was not clear. There was a positive association between pack-years of cigarette smoking and the risk of rectal cancer in men. A positive dose–response relation between alcohol consumption and colon cancer risk was observed for men and women.

Colorectal cancer has become the third leading cause of cancer-related mortality in Japan (Ministry of Health, Labor and Welfare, 1999), and its incidence has been increasing more rapidly than in other industrialised countries for several decades (WCRF, AIRC, 1997). This appears to be related to lifestyle changes, such as, for example, diet.

Body size, as well as diet, has been investigated in relation to the occurrence of the cancer. Height is affected not only by genetic factors, but also by nutritional condition during childhood and adolescent. Some studies (Albanes *et al*, 1988; Chute *et al*, 1991; Bostick *et al*, 1994; Giovannucci *et al*, 1995; Robsahm and Tretli, 1999; Smith *et al*, 2000), but not all (Okasha *et al*, 2000), have reported a positive association between height and colon cancer. Results of studies on body mass index (BMI) in relation to colorectal cancer have been also inconsistent. There are relatively few prospective studies of colorectal cancer among Japanese (Nomura *et al*, 1985; Kono *et al*, 1987; Hirayama, 1989; Akiba, 1994), and only Nomura *et al* (1985) investigated BMI, which was positively associated with an increased risk for colon cancer among Japanese male immigrants to Hawaii. We have therefore conducted a prospective study, among Japanese people, of colorectal cancer in relation to height, BMI, alcohol consumption, and smoking habit.

## MATERIALS AND METHODS

A cohort was established in September 1992 with residents in Takayama, Japan, who were 35 years old or older (Shimizu, 1996). A self-administered questionnaire covering sociodemographic characteristics, drinking, smoking, diet, exercise, and reproductive and medical histories was distributed to 36 990 residents, of whom 34 018 (92.0%) responded. Subjects who left four out of nine two-page spreads of questionnaire or more all blank (*n*=595, 1.7%), and those who inadequately reported to the questionnaire (*n*=1871, 5.5%) were excluded from the cohort. The fixed cohort consisting of 31 152 subjects was defined.

The 1992 questionnaire sought details of current height, weight, and weight at age 21 years. The intraclass correlation coefficients between self-reported and measured height and weight in a subsample were 0.93 and 0.97 in both sexes, respectively.

Diet was assessed by a semiquantitative food–frequency questionnaire that contained 169 food items, covering average consumption frequencies and serving sizes of selected food items during the previous year. Individual nutrient intake was estimated based upon the frequency of intake and portion size using the *Standard Tables of Food Composition in Japan*, 5th edition, published by the Science and Technology Agency of Japan. Details including results of validity tests are described elsewhere (Shimizu *et al*, 1999).

The questions on alcohol use included six types, that is, sake, beer, light beer, shochu (distilled from sweet potatoes, rice, or buckwheat), wine, and hard liquor. For each item, the questionnaire included nine frequency categories (never/less than once a month; once a month; twice or three times a month; once a week; twice or three times a week; four to six times a week; once a day; twice a day; more than four times a day) and the number of cups, glasses, and bottles consumed. The amount of ethanol was calculated in grams using the *Standard Tables* mentioned above. The correlation coefficients that compare alcohol consumption estimated from the questionnaire with 12 one-day diet diaries at about 1-month intervals over 1 year were 0.72 and 0.64, for men and women, respectively (data not published).

Years of smoking and the number of cigarettes smoked each day were reported. Those who had smoked a total of 20 or more packs of cigarettes in their lifetime were defined as smokers.

Physical activity was based on average hours per week spent performing various kinds of activities; details, including the results of the validity tests, are described elsewhere (Suzuki *et al*, 1998; Shimizu, 2001).

Participants who did not report their height (564 men, 709 women) were excluded from the study, as were those who reported cancer other than nonmelanoma skin cancer (173 men, 532 women) or colorectal adenoma (281 men, 208 women) at baseline. We could not obtain information about diagnosis for 13 men and 15 women who were known to have died of colorectal cancer; thus, these men and women were also excluded from the analysis. The subjects totalled 29 051(13 392 men and 15 659 women).

There were 198 (105 men, 93 women) with colon and 97 (56 men, 41 women) with rectal cancer, all diagnosed histologically at two major hospitals in Takayama City during the follow-up from 1 January 1993 to 31 December 2000. Compared to the number of colorectal cancer occurred in the city according to the annual reports issued by the prefecture government (Hida Public Health Center, 2000), the two hospitals covered about 90% of colorectal cancers in the city each year. Details of subjects who moved away from the city during the study period were obtained from the residential registers, namely, 629 (4.7%) men and 508 (3.2%) women.

We analysed height, BMI, and smoking and drinking habits in relation to colorectal cancer risk with a separate analysis for colon and rectal cancers. Individuals were categorised into tertiles according to the distribution of height and BMI. Variables for smoking and drinking were categorised, and tests for a linear trend were performed on ordinal variables or continuous variables with the use of median values of the category. Person-years were accumulated up to death, loss to follow-up or the end of 2000, whichever occurred first. Cox proportional hazard models were used to calculate hazard ratios. All reported *P*-values were two sided. In all proportional hazard models, adjustments were made for age, and, where indicated in the text and tables, for other known risk factors. All statistical analyses were performed using PC-SAS (SAS Institute, SAS/STAT user's guide, Version 8.2, SAS Institute, Cary, NC, USA).

## RESULTS

The mean ages (standard deviations) of participants were 54.1 (12.2) for men and 55.1 (13.0) for women. The average heights of each age strata were comparable to those of the general population in Japan. The distribution of various factors possibly related to colorectal cancer by height and sex is shown in Table 1

**Table 1:** Distribution of various factors by sex and height

	**Height (cm)**					
	**Men**			**Women**		
	**Short**	**Medium**	**Tall**	**Short**	**Medium**	**Tall**
	**⩽162**	**163–167**	**⩾168**	**⩽150**	**151–154**	**⩾155**
Number	4863	3932	4597	6393	3643	5623
Age (years)^a^	60.2 (11.9)	53.8 (11.0)	48.0 (10.0)	61.9 (13.0)	53.2 (10.9)	48.8 (10.4)
Height (cm)^a^	157.5 (4.41)	164.9 (1.37)	171.7 (3.46)	146.0 (4.51)	152.6 (1.03)	158.3 (3.12)
Weight (kg)^a^	55.4 (7.43)	61.1 (7.46)	67.1 (8.80)	47.1 (6.99)	51.4 (6.58)	54.7 (7.12)
Body mass index (kg m^−2^)^a^	22.3 (2.87)	22.5 (2.72)	22.7 (2.79)	22.1 (3.14)	22.1 (2.80)	21.8 (2.76)
Diabetes mellitus (%)	7.1	6.5	5.0	3.6	2.7	1.8
Cholecystectomy (%)	1.2	0.7	0.7	1.2	1.5	0.8
Education <12 years (%)	74.9	56.7	61.4	83.1	64.2	48.4
Physical activity score (METs^b^–hour week^−1^)^a^	26.5 (40.0)	27.8 (41.5)	29.4 (44.2)	16.8 (28.1)	20.7 (30.4)	22.6 (32.0)
Total energy intake (kcal day^−1^)^a^	2468 (839)	2610 (874)	2723 (880)	2006 (761)	2169 (789)	2230 (576)
Alchol intake (g day^−1^)^a^	36.9 (38.7)	42.7 (41.9)	44.7 (42.8)	6.18 (14.2)	7.48 (16.3)	9.54 (19.3)
Ever-smokers (%)	81.5	83.2	85.2	14.6	16.5	20.8

a Values are means (s.d.).

b METs=metabolic equivalents.

. Age varied inversely with height. The percentages of ever-smokers and persons with higher education increased across the strata of height. Taller men and women consumed more calories and were more physically active. The incidence rates in this cohort by age strata were comparable to those in the general population in Japan (The Research Group for Population-based Cancer Registration in Japan, 2002).

Positive relations between height and colon cancer are seen for men, but were weaker and nonsignificant for women (Table 2

**Table 2:** Relative risks for colorectal cancer according to height

	**Men, height (cm)**			
	**Low, ⩽162**	**Medium, 163–167**	**High, ⩾168**	***P* for trend**
*Colon cancer*	
Person-years	36 584	30 108	35 315	
Number	36	36	36	
Age-adjusted RRs (95% CI)	1.00	1.73 (1.08–2.78)	2.09 (1.26–3.46)	0.003
Multivariate RRs (95% CI)	1.00	1.75 (1.07–2.85)	2.13 (1.26–3.58)	0.004
				
*Rectal cancer*				
Person-years	36 527	30 064	35 190	
Number	23	24	12	
Age-adjusted RRs (95% CI)	1.00	1.91 (1.06–3.45)	1.23 (0.58–2.60)	0.31
Multivariate RRs (95% CI)	1.00	1.87 (1.02–3.44)	1.21 (0.57–2.61)	0.37
				
	**Women, height (cm)**			
	**Low, ⩽150**	**Medium, 151–154**	**High, ⩾155**	***P* for trend**
*Colon cancer*				
Person-years	43 749	29 299	39 673	
Number	43	25	25	
Age-adjusted RRs (95% CI)	1.00	1.50 (0.88–2.56)	1.44 (0.84–2.48)	0.15
Multivariate RRs (95% CI)	1.00	1.56 (0.87–2.81)	1.48 (0.81–2.70)	0.18
				
*Rectal cancer*				
Person-years	43 658	25 588	39 596	
Number	19	10	12	
Age-adjusted RRs (95% CI)	1.00	1.10 (0.49–2.45)	1.15 (0.51–2.59)	0.74
Multivariate RRs (95% CI)	1.00	1.27 (0.53–3.06)	1.30 (0.52–3.21)	0.56

Multivariate=age, BMI, alcohol consumption, smoking, and years of education. CI=confidence interval.

). For men, the relative risks (RRs) of colon cancer from the shortest to the middle and tallest height categories were 1.75 (95% confidence interval, CI=1.07–2.85) and 2.13 (95% CI=1.26–3.58), respectively, with a *P* for trend of 0.004, after controlling for age, BMI, alcohol consumption, smoking, and education. For women, the point estimates of colon cancer risk for the middle and the tallest categories of height were moderately but not significantly elevated. Further adjustments for physical activity did not substantially alter the results (RRs of colon cancer for the tallest height tertile compared with the shortest tertile adjusted for age, BMI, alcohol consumption, smoking, education, and physical activity=2.07 (95% CI=1.21–3.52) and 1.56 (95% CI=0.85–2.88), for men and women, respectively). We further performed adjustments for nutrient intake, such as total energy and dietary fibre. The results were not modified by the adjustments.

Table 3

**Table 3:** Relative risks for colorectal cancer according to BMI

	**Men, BMI (kg m^−2^)**			
	**⩽21.2**	**21.3–22.4**	**⩾23.6**	***P* for trend**
*Colon cancer*	
Person-years	32 685	33 839	33 772	
Number	26	35	43	
Age-adjusted RRs (95% CI)	1.00	1.47 (0.88–2.44)	1.98 (1.21–3.25)	0.007
Multivariate RRs (1) (95% CI)	1.00	1.46 (0.87–2.46)	2.07 (1.26–3.42)	0.004
Multivariate RRs (2) (95% CI)	1.00	1.57 (0.92–2.68)	2.11 (1.26–3.53)	0.005
				
*Rectal cancer*				
Person-years	32 669	33 687	33 728	
Number	24	18	16	
Age-adjusted RRs (95% CI)	1.00	0.85 (0.46–1.56)	0.85 (0.45–1.60)	0.60
Multivariate RRs (1) (95% CI)	1.00	0.76 (0.40–1.44)	0.79 (0.41–1.52)	0.47
Multivariate RRs (2) (95% CI)	1.00	0.80 (0.41–1.55)	0.83 (0.42–1.64)	0.59
				
	**Women, BMI (kg m^−2^)**			
	**⩽21.6**	**21.7–23.0**	**⩾23.1**	***P* for trend**
*Colon cancer*				
Person-years	35 100	35 905	36 373	
Number	28	25	36	
Age-adjusted RRs (95% CI)	1.00	1.06 (0.61–1.82)	1.14 (0.68–1.90)	0.62
Multivariate RRs (1) (95% CI)	1.00	1.08 (0.58–2.00)	1.26 (0.72–2.22)	0.39
Multivariate RRs (2) (95% CI)	1.00	1.02 (0.55–1.91)	1.22 (0.69–2.15)	0.48
				
*Rectal cancer*				
Person-years	25 046	35 836	36 290	
Number	14	12	15	
Age-adjusted RRs (95% CI)	1.00	0.77 (0.35–1.71)	0.92 (0.44–1.92)	0.86
Multivariate RRs (1) (95% CI)	1.00	0.86 (0.36–2.02)	0.98 (0.44–2.20)	0.99
Multivariate RRs (2) (95% CI)	1.00	0.84 (0.36–1.99)	0.83 (0.35–1.99)	0.68

Multivariate (1)=adjusted for age, height, alcohol consumption, smoking, and years of education. Multivariate (2)=adjusted for age, height, alcohol consumption, smoking, years of education, and physical activity. CI=confidence interval.

shows that BMI was positively associated with the risk of colon cancer for men, whereas the association for women was weak, controlling for age, height, alcohol consumption, smoking, and education. There was no significant association between height and the risk of rectal cancer in both sexes. Further adjustment for physical activity did not substantially alter the results. Our additional analysis for BMI calculated using self-reported weight at age 21 years did not demonstrate any associations or trends (data not shown).

The associations between total alcohol intake and colorectal cancer are shown in Table 4

**Table 4:** Relative risks for colorectal cancer according to alcohol consumption

	**Men, alcohol intake**			
	**No alcohol**	**⩽36.7 g day^−1^**	**>36.7 g day^−1^**	***P* for trend**
*Colon cancer*	
Person-years	8088	46 494	47 424	
Cases, *n*	5	45	58	
Age-adjusted RRs (CI)	1.00	1.94 (0.77–4.90)	2.96 (1.17–7.46)	0.007
Multivariate RRs (CI)	1.00	1.79 (0.71–4.55)	2.67 (1.06–6.76)	0.01
				
*Rectal cancer*				
Person-years	8114	46 379	47 289	
Cases, *n*	8	20	31	
Age-adjusted RRs (CI)	1.00	0.57 (0.25–1.30)	1.12 (0.50–2.51)	0.06
Multivariate RRs (CI)	1.00	0.59 (0.25–1.42)	1.17 (0.50–2.73)	0.06
				
	**Women, alcohol intake**			
	**No alcohol**	**⩽3.75 g day^−1^**	**>3.75 g day^−1^**	***P* for trend**
*Colon cancer*				
Person-years	36 265	36 394	36 416	
Cases, *n*	34	28	32	
Age-adjusted RRs (CI)	1.00	0.87 (0.51–1.49)	1.52 (0.92–2.53)	0.04
Multivariate RRs (CI)	1.00	1.07 (0.58–1.96)	1.78 (1.00–3.18)	0.03
				
*Rectal cancer*				
Person-years	36 131	36 325	36 385	
Cases, *n*	7	15	19	
Age-adjusted RRs (CI)	1.00	1.88 (0.74–4.79)	2.27 (0.91–5.69)	0.17
Multivariate RRs (CI)	1.00	1.20 (0.44–3.26)	1.80 (0.70–4.62)	0.17

Multivariate=adjusted for age, height, BMI, smoking, and years of education.

. Relative risks of colon cancer for individuals in the highest alcohol consumption compared with abstainers were 2.52 (95% CI=1.00–6.38) and 1.73 (95% CI=0.98–3.05) for men and women, respectively, controlling for age, height, BMI, smoking, and education. However, a significant positive relation with rectal cancer risk was restricted to women. In the analysis by type of beverage, sake consumption was significantly associated with colon cancer risk in men (the RR from the abstainers to the highest sake consumption category=1.91; 95% CI=1.10–3.32).

Men smoked more than 20 pack-years of cigarettes were associated with elevated rectal cancer risk (Table 5

**Table 5:** Relative risks for colorectal cancer according to tobacco smoking

	**Men, smoking status**			
	**Never smoked**	**Smokers ⩽20 pack-years**	**Smokers >20 pack-years**	***P* for trend**
*Colon cancer*	
Person-years	16 702	42 141	37 361	
Cases, *n*	16	41	47	
Age-adjusted RRs (CI)	1.00	1.33 (0.78–2.28)	1.43 (0.85–2.41)	0.09
Multivariate RRs (CI)	1.00	1.36 (0.79–2.33)	1.37 (0.81–2.32)	0.19
				
*Rectal cancer*				
Person-years	16 653	42 009	37 327	
Cases, *n*	7	16	34	
Age-adjusted RRs (CI)	1.00	1.22 (0.54–2.78)	2.36 (1.13–4.92)	0.03
Multivariate RRs (CI)	1.00	1.33 (0.57–3.12)	2.44 (1.12–5.30)	0.04
				
	**Women, smoking status**			
	**Never smoked**	**Smokers ⩽10 pack-years**	**Smokers >10 pack-years**	***P* for trend**
*Colon cancer*				
Person-years	82 430	8944	7273	
Cases, *n*	68	4	5	
Age-adjusted RRs (CI)	1.00	0.65 (0.24–1.76)	0.97 (0.39–2.40)	0.91
Multivariate RRs (CI)	1.00	0.59 (0.21–1.62)	0.77 (0.30–1.96)	0.54
				
*Rectal cancer*				
Person-years	82 266	8944	7264	
Cases, *n*	32	4	2	
Age-adjusted RRs (CI)	1.00	1.69 (0.59–4.86)	1.06 (0.25–4.47)	0.47
Multivariate RRs (CI)	1.00	1.76 (0.60–5.14)	0.94 (0.21–4.16)	0.63
Multivariate=adjusted for age, height, BMI, alcohol intake, and years of education.				

). No pattern of changing risk was observed in women who smoked more than 10 pack-years.

## DISCUSSION

These data indicated that being tall or overweight is associated with an increased risk of colon cancer in men, while alcohol consumption was associated with colon cancer risk in both sexes.

Six other prospective studies (Albanes *et al*, 1988; Chute *et al*, 1991; Bostick *et al*, 1994; Giovannucci *et al*, 1995; Robsahm and Tretli, 1999; Smith *et al*, 2000), but not all, support our finding that being tall significantly elevates the risk of colorectal cancer. One possibility is that taller people may have longer intestines (Hirsch *et al*, 1956) and have a greater rate of cell division within the tissue (Albanes and Winick, 1988); thus, more colon cells may be at risk. Greater exposure to mitogenic factors, such as growth hormone, insulin, insulin-like growth factors, and sex steroids, could also result in increased cancer risk. Height has been related to elevated risk of several specific cancers, including breast (Swanson *et al*, 1988) and prostate (Smith *et al*, 2000). Animal experiments have shown that low-energy diets from early age shorten overall animal length and reduce cancer risk (Kritchevsky, 1995). In humans, wartime food deprivation for pubescent women was associated with lower breast cancer rates than in younger and older cohorts (Tretli and Gaard, 1996). Furthermore, an association has been reported between higher caloric intake during childhood and higher rates of cancer (Frankel *et al*, 1998). Height is partly determined by total caloric intake during childhood and adolescence; so its association with colon cancer may indicate that total caloric intake during childhood is relevant for a later colon cancer risk.

Evidence has long suggested that people with excess body weight are at higher risk of colorectal cancer (Lew and Garfinkel, 1979; Nomura *et al*, 1985; Albanes and Taylor, 1990). Our study, as well as others (Garfinkel, 1985; Wu *et al*, 1987; Russo *et al*, 1998; Robsahm and Tretli, 1999; Murphy *et al*, 2000), shows a positive association between the risk of colon cancer of BMI in men but not in women. Other case–control studies (Slattery *et al*, 1997; Caan *et al*, 1998) and a cohort study (Ford, 1999) have shown a significant positive association between the risk of colon cancer and BMI in both sexes, although this was weaker among women in the two case–control studies. Central obesity, which may increase colon cancer risk by acting as a tumour-growth promoter or mitogen (Giovannucci, 1995; Bjorntorp, 1991), is more common among males. Thus, BMI may simply be a more accurate indicator of central obesity for men than for women (Murphy *et al*, 2000). Another possibility is a protective effect of oestrogen.

Our study also shows a significant positive dose–response relation between alcohol consumption and colon cancer risk in both sexes, although not significant in women as found in other studies. In a prospective study in California, the RR of colorectal cancer was 2.42 (95% CI=1.3–4.5) in men who drank more than 30 ml day^−1^ compared to nondaily alcohol drinkers, while the RR among women who consumed more than 30 ml day^−1^ relative to the same group was weaker and not significant (RR=1.45, CI=0.8–2.6) (Wu *et al*, 1987). Similar results were obtained when the cases of rectal cancer were omitted. Klatsky *et al* (1988) prospectively studied and found a positive relation between the risk of colon cancer and total alcohol intake in both sexes, although the results in women but not men were statistically significant. On the other hand, a meta-analysis (Longnecker *et al*, 1990) including both prospective and case–control studies showed that the RR of colorectal cancer was 1.10 (95% CI=1.05–1.14), and the association did not vary according to gender or site within the large bowel. In almost all case–control studies, the use of hospital controls, among which proportions of alcohol-related disease were high, may have biased the results.

There was no association between BMI and rectal cancer risk in both the sexes. Similar finding was obtained in a case–control study reported by Dietz *et al* (1995), that body weight was positively associated with colon cancer but not with rectal cancer. Positive association between BMI and rectal cancer has been reported in some studies (Phillips and Snowdon, 1985; Russo *et al*, 1998), but not in others (Dietz *et al*, 1995; Howe *et al*, 1997). A significant association between alcohol consumption and rectal cancer risk has been reported (Klatsky *et al*, 1988; Hirayama, 1989), but in our study, the association was weak in men. We must mention that the number of cases of rectal cancer might be too small to have meaningful analyses.

Recent prospective studies have suggested a positive association between long-term and heavy smoking and colon cancer (Giovannucci *et al*, 1994; Hsing *et al*, 1998; Terry *et al*, 2001). In our present study, no significant association was observed in relation to the quantity of cigarettes smoked except for rectal cancer risk in men.

A major advantage of our study is that it is population based and prospective, thereby minimising recall bias. Incidence rather than mortality can avoid bias because of a progress of medical treatment as well as effect of disease. Furthermore, the data were analysed adjusting for physical activity, nutrition, and years of education.

However, several limitations must be considered. Firstly, height was self-reported and not measured. Although the intraclass correlation coefficient between self-reported height and measured height in a subsample was relatively high, self-reported information may result in misclassification. However, since the height information was collected before the development of cancer, any misclassification would be nondifferential with respect to the disease. Such random misclassification would tend to attenuate RR estimates, and, thus, would not explain the association between height and colon cancer.

Secondly, height was reported at the baseline. Stature has been reported to be at its peak at the end of the third decade of life (Friedlander *et al*, 1977). Our subjects were 35 years old or over, so their height at the baseline might have been already reduced by ageing or disease and may not have represented their highest. However, age was adjusted for in our analysis, while additional adjustment for pre-existing disease, such as hypertension and diabetes mellitus, did not substantially alter the results.

Another limitation is incomplete detection of colorectal cancers, although details from the two study hospitals appear to cover around 90% of the colorectal cancers in the city reported by the prefectural cancer registry. Some colorectal cancer patients must therefore have been classified as subjects without colorectal cancer. However, it is unlikely that taller or heavier patients were selectively included in the present study. In addition, the study was so large that the effect of misclassification of true cases was minimal.

In summary, this cohort study provides evidence that height and overweight may be associated with an increased risk of colon cancer for Japanese men, and heavy alcohol consumption may increase the risk of colon cancer in both sexes.
